# Observation of Complete Orbital Two-Channel Kondo Effect in van der Waals Ferromagnet Fe_3_GaTe_2_

**DOI:** 10.3390/nano16020123

**Published:** 2026-01-16

**Authors:** Chunhao Bao, Zhiyue Li, Xiaolong Yin, Jifeng Shao, Longxiang Li, Xiaoming Ma, Shu Guo, Tingyong Chen

**Affiliations:** 1 Department of Physics, Southern University of Science and Technology, Shenzhen 518055, China; 2 Shenzhen Institute for Quantum Science and Engineering, Southern University of Science and Technology, Shenzhen 518055, China; 3 International Quantum Academy, Shenzhen 518048, China

**Keywords:** Fe_3_GaTe_2_, orbital two-channel Kondo effect, magnetotransport

## Abstract

The orbital two-channel Kondo (2CK) effect is one of the crucial systems with non-Fermi liquid (NFL) behavior. But the full three-regime transport evidence has never been observed in one sample. Here, all three resistive regimes for the orbital 2CK effect induced by two-level systems (TLSs) have been observed in the van der Waals ferromagnet Fe_3_GaTe_2_. Electron behavior undergoes a continuous transition from electron scattering to NFL behavior, and subsequently to Fermi liquid behavior. The magnetic field does not affect any regimes, indicating the nonmagnetic origin of the TLSs in Fe_3_GaTe_2_. In addition, instead of topological Hall, the slope of a linear negative magnetoresistance is related to spin-magnon scattering and could be utilized to infer the emergence of spin textures. Our findings indicate that Fe_3_GaTe_2_ may be an ideal platform to study electron correlation and topological phenomena.

## 1. Introduction

The metallic state of many solids can be understood in the framework of Landau’s Fermi liquid theory (FLT), where dynamics at low excitation energies and temperatures are described by substituting the non-interacting fermions with interacting quasiparticles carrying the same spin, charge and momentum [1]. However, some of the most intriguing phenomena in strongly correlated systems lie beyond the quasiparticle paradigm, where quantum criticalities may provide a better understanding [2]. Examples of such non-Fermi liquids (NFLs) include Luttinger liquids [3,4,5], fractional quantum Hall Laughlin liquids [6], high-temperature superconductors [7], heavy fermions [8,9] and the two-channel Kondo (2CK) system [10,11]. Although the Kondo ground state is complex, its excitations can still be described by FLT. In the spin 2CK effect, however, a spin-1/2 impurity couples to conduct electrons into two equal orbital channels and leads to impurity quantum criticality with exotic NFL behavior as a consequence of two spins attempting to compensate the spin-1/2 impurity. But the spin 2CK effect is difficult to observe because of the strict requirements of zero local magnetic field and channel symmetry. Instead, an analogous orbital 2CK effect was proposed based on resonant scattering centers with orbital degrees of freedom such as two-level systems (TLSs) [12,13,14]. In this scenario, the TLS assumes a role equivalent to that of the impurity spin-1/2 in the spin 2CK effect and is thus termed a pseudo-spin-1/2 [12,15]. Due to the larger orbital degree of freedom inherent in the TLSs, the orbital 2CK effect is supposed to be more readily observable.

The 2CK effect has attracted significant attention due to its relevance to high-temperature superconductivity [1], Majorana fermions [2], and strongly correlated physics [3]. The hallmark features of the orbital 2CK effect are the three-resistive regimes in upturn resistance at low temperature: (1) Kondo regime: *T*_K_ < *T* < *T*_0_, *R*_xx_ ~ −ln(*T*), where the weak coupling starts at *T*_0_ and ends at the Kondo Temperature (*T*_K_) due to electron scattering with the TLSs. (2) NFL regime: *T*_D_ (=∆^2^/*T*_K_) < *T* ≪ *T*_K_, *R*_xx_ ~ −*T*^1/2^, electrons compete to screen the pseudo-spin-1/2 and contribute to the “over screened” of the scattering center [16,17]. This regime is absent in the Kondo effect and ∆ is the energy splitting between the localized states. (3) Fermi liquid (FL) regime: *T* < *T*_D_, *R*_xx_ ~ −*T*^2^, where the FL state shows up due to complete screening of the pseudo-spin-1/2. Because of the nonmagnetic origin of the TLSs, the three-resistive regimes do not depend on external magnetic fields.

Previously, the resistance upturn has been observed in many systems, including Cu point contact [13,18,19], glasslike ThAsSe [20], epitaxial ferromagnetic *L*1_0_-MnAl films [21,22] and layered compound ZrAs_1.58_Se_0.39_ [23]. In addition, van der Waals (vdWs) ferromagnets such as Fe_3_GeTe_2_ (FGeT) also show the characteristics of resistance upturn at low temperatures [24,25]. These effects are attributed to the orbital 2CK effect, but are not without controversies [12,16,26,27], because the three-resistive regimes expected from the orbital 2CK effect are not fully observed in a single material sample. Furthermore, NFL phenomena in vdWs materials are expected to deviate strongly from FL [28]. Ferromagnetic Fe_3_GaTe_2_ (FGaT) is a unique vdWs material which has a Curie temperature (*T*_C_) of 340 K, above room temperature [29]. Since ferromagnetic thin films, such as *L*1_0_-MnAl [21], *L*1_0_-MnGa [30] and FGeT [31], have already shown electrical transport characteristics associated with the orbital 2CK effect, FGaT may represent an ideal vdWs material platform to study the orbital 2CK effect.

In this paper, we have observed the hallmark feature of the three-resistive regimes of temperature-dependence in a single FGaT material system, which is possible evidence for the orbital 2CK effect. The longitudinal resistance *R*_xx_ undergoes a transformation across three consecutive temperature regimes, from electron-TLS scattering (~−ln(*T*): 30 K–9 K) to NFL behavior (~−*T*^1/2^: 9 K–1 K), then finally the FL behavior (~−*T*^2^: <1 K) due to complete screening of the pseudo-spin-1/2. The fact that magnetic fields up to 9 T do not disrupt the three-regimes behavior indicates that the orbital 2CK effect in FGaT originates from nonmagnetic TLSs. The topological Hall effect is not observed and the antisymmetric Hall peaks at room temperature are due to the anomalous Hall effect. We have found out that the linear negative magnetoresistance (LNMR) can be useful to characterize spin textures in magnetic materials. For decreasing temperature, the slope of the LNMR of the FGaT sample does not decrease monotonically due to the increase in the spin-magnon scattering around 130 K.

## 2. Materials and Methods

### 2.1. Crystal Growth

The FGaT single crystals were grown using the self-flux method. Fe powder (99.99%, Macklin, Shanghai, China), Ga ingots (99.98%, Macklin, Shanghai, China), and Te powder (99.99%, Macklin, Shanghai, China) were homogeneously mixed in a molar ratio of 1:1:2 within a glove box and subsequently sealed in a vacuum-sealed quartz tube. The mixture was rapidly heated to 1000 °C within 1 h and maintained at 1000 °C for 24 h. Subsequently, the temperature rapidly decreased to 780 °C within 1 h and was held at 780 °C for 100 h. Finally, the sample was subjected to a temperature-controlled centrifugation to separate the FGaT crystals from the flux.

### 2.2. Structure Characterizations

X-ray diffraction measurements were conducted on the FGaT sample with a Rigaku Smartlab 3K (Rigaku, Tokyo, Japan), yielding the (00*l*) diffraction peaks [32]. Subsequently, the elemental composition of the FGaT samples was qualitatively measured and elemental mapping images were acquired using an energy dispersive spectrometer (EDS, Aztec XmaxN 50, Oxford Instruments, Concord, MA, USA) [33]. A single-crystal X-ray diffractometer (Bruker D8 VENTURE; Bruker, Billerica, MA, USA) was utilized to determine the precise elemental composition and ratios, and diffraction images of the FGaT single-crystal samples were obtained.

### 2.3. Device Fabrication and Transport Measurement

The high-quality single crystal FGaT was mechanically exfoliated with silicon-free blue tape and transferred onto SiO_2_/Si substrate. The standard hall bar electrode was patterned by a laser direct writing machine (DWL 66+) and then coated with Ti/Au (5 nm/50 nm) using an electron beam evaporation coating system (JEB-2). All transport measurements were carried out in a physical property measurement system (PPMS DynaCool; Quantum Design, San Diego, CA, USA) with a base temperature of 1.8 K and a magnetic field of up to 14 T. Additionally, the Helium-3 cryostat accessory for PPMS enables the measurement of temperatures down to 500 mK.

## 3. Results

**Figure 1:**
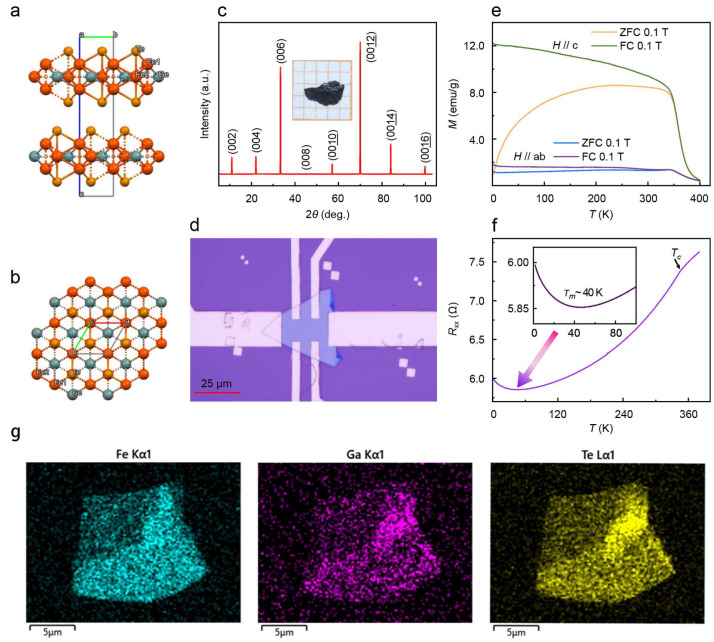
Crystal structure and transport characterization of thin FGaT flakes. (**a**) Atomic structure of an FGaT bilayer. Fe(I) and Fe(II) indicate the two distinct Fe site with +3 and 0 valence states. (**b**) Top view of FGaT. (**c**) XRD pattern of newly cleaved single crystal FGaT. The inset shows the optical image of the FGaT single crystal. (**d**) Optical image of the FGaT nanoflake Hall bar for transport measurement. Sample thickness: 81.4 nm. (**e**) Temperature dependence of magnetization during zero-field-cooling (ZFC) and field-cooling (FC) processes for *H*∥*c* and *H*∥*ab* at *µ*_0_*H* = 0.1 T. (**f**) Temperature dependence of longitudinal resistance *R*xx. The inset shows the resistance upturn around 40 K. (**g**) EDS mapping images of thin FGaT flakes. The letters a, b, and c in subfigures (**a**,**b**) represent the crystallographic axes (a-axis, b-axis, and c-axis) of the unit cell.

The ferromagnet FGaT single crystals were synthesized by the self-flux method. The single-crystal XRD pattern confirms a hexagonal structure with the space group P6_3_/mmc (*a* = *b* = 4.0767 Å, *c* = 16.088 Å). The high quality of the crystal is further substantiated by single-crystal refinement, yielding excellent reliability factors (R_1_ = 0.0347, wR_2_ = 0.0827) and a Goodness-of-Fit of 1.1595. The XRD pattern exhibits a typical (00*l*) orientation with extremely narrow peak widths, indicating the high crystallinity of the as-grown material. In each layer of FGaT, covalently bonded Fe_3_Ga contains the hexagonal Fe(I)-Ga atomic ring layer and two separated triangular Fe(I)-Fe(I) lattice layers, and both are sandwiched by two adjacent atomic layers with weak vdWs interlayer coupling, as shown in Figure 1a,b. The XRD pattern of the FGaT single crystal in Figure 1c with all (00*l*) Bragg peaks indicates that the c-axis is perpendicular to the newly cleaved surface of *ab*-plane. Following mechanical exfoliation, the FGaT thin films are transferred onto substrates and further patterned as the transport measurement structure, as illustrated in Figure 1d. Figure 1e is the temperature-dependent magnetization of the FGaT single crystal. These measurements were conducted under conditions of ZFC and FC, with a magnetic field of 0.1 T applied along both the c-axis and the *ab*-plane separately. The Curie temperature (*T*_C_) is around 340 K. The splitting of ZFC and FC curves indicates the formation of a multidomain at low temperature. The field-cooled magnetization measured with *H*∥*c* is much larger than that of *H*∥*ab*, revealing the out-of-plane easy axis of the FGaT crystal flake. Figure 1f shows the temperature-dependence of in-plane longitudinal resistance *R*_xx_ cooling from 380 K to 1.8 K at *µ*_0_*H* = 0 T with a current in the *ab*-plane. The resistance curve shows a clear kink at *T*_C_ around 340 K. Subsequently, a minimum in the longitudinal resistance occurs around 40 K, followed by an abnormal upturn as the temperature decreases. This upturn is a potential signature of the Kondo effect. The following detailed analysis of its temperature-dependence provides compelling evidence that it originates specifically from the orbital two-channel Kondo (2CK) effect.

**Figure 2:**
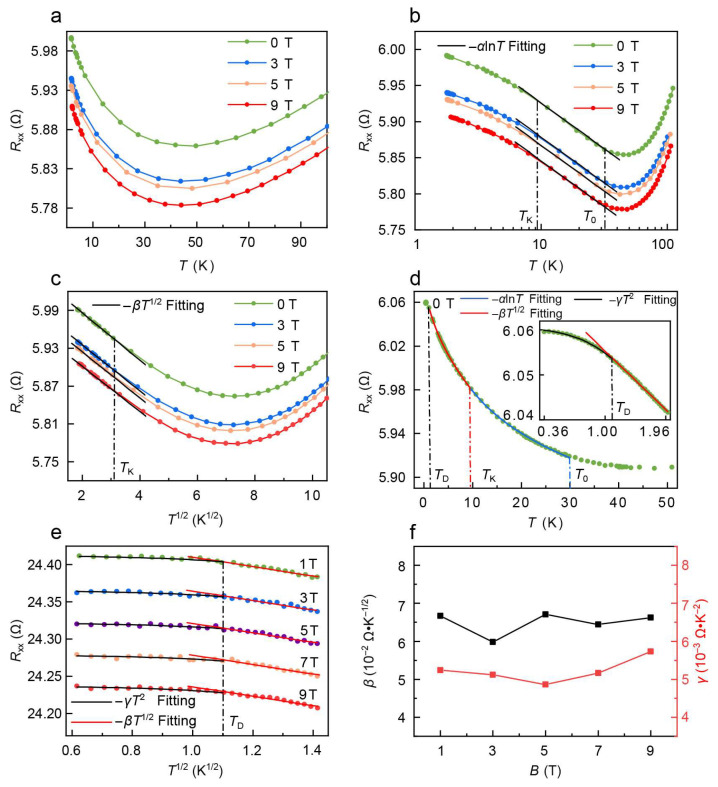
Evidence for orbital 2CK effect and Fermi liquid behavior down to 0.36 K. (**a**) The longitudinal resistance *R_xx_* vs. *T* at different magnetic fields. (**b**) Semilog plot of the longitudinal resistance *R_xx_* vs. ln(*T*) under different fields. The dotted lines are experimental data. (**c**) The longitudinal resistance *R_xx_* vs. *T*^1*/*2^ under different fields. The dotted lines are experimental data. (**d**) Temperature-dependent *R_xx_* from 50 K to 0.36 K under no external field. The solid dots are experimental data. The inset shows the longitudinal resistance *R_xx_* from 2 K to 0.36 K at *µ*_0_*H* = 0 T. (**e**) The longitudinal resistance *R_xx_* vs. *T*^1*/*2^ from 2 K to 0.36 K under different magnetic fields after the sample was oxidized in an atmosphere for 7 days. The dotted lines are experimental data. (**f**) The variation in the slope parameters *β* and *γ* with the magnetic field.

To further reveal the mechanism behind the resistance upturn in FGaT, we measured the temperature-dependence of the longitudinal resistance under different magnetic fields up to 9 T. As shown in Figure 2a, the resistance minimum remains at the same temperature in the magnetic field, and the resistance upturn exhibits no sign of change except a vertical shift with magnetic fields up to 9 T. The persistence of resistance upturn under a magnetic field suggests that the weak localization effect will not be responsible for the resistance upturn in the FGaT system [34]. To characterize the evolution of temperature-dependent resistance, the experimental data is the most well-fitted with −*α*ln(*T*), −*βT*^1/2^, and −*γT*^2^; the results are plotted in Figure 2b–d. *α*, *β* and *γ* are the coefficients of the theoretical fitting line, respectively. It shows that the *R*_xx_(*T*) exhibits an apparent crossover from −*α*ln(*T*) dependence to −*βT*^1/2^ dependence, as shown in the linear region of Figure 2b,c. For temperatures between 30 K and 9 K, *R*_xx_(*T*) gradually increases, following a strong −*α*ln(*T*) dependence. Obviously, the data show no characteristic transition from a −*α*ln(*T*) dependence to a −*γT*^2^ dependence, suggesting that the single-channel Kondo effect is not the dominant mechanism in our FGaT system. As the temperature subsequently drops, a strong −*βT*^1/2^ dependence emerges and fits well down to 1.8 K, as shown in Figure 2c. The −*βT*^1/2^ dependence cannot be attributed to electron–electron interaction (EEI), as EEI typically manifests at very low temperatures in the millikelvin range [35]. However, the observed −*βT*^1/2^ dependence dominates up to 9 K but diminishes below 1 K. This excludes the possibility that the quantum interference effects typically encountered in disorder systems, including EEI and localization, contribute to quantum corrections in the *R*_xx_(*T*) behavior [36]. The observed crossover from a linear −*α*ln(*T*) dependence to a −*βT*^1/2^ dependence suggests that the −*βT*^1/2^ dependence should be attributed to the NFL behavior associated with the orbital 2CK effect [37]. To determine the *R*_xx_(*T*) at further lower temperatures, we measured *R*_xx_ from 1.8 K down to 0.36 K for more details. As shown in Figure 2e, the −*βT*^1/2^ trend persists down to approximately 1 K. Below 1 K, the *R*_xx_(*T*) deviates from −*βT*^1/2^, and exhibits a clear saturation down to 0.36 K.

As shown in Figure 2, the Kond regime in the FGaT sample starts at *T*_0_ ~ 30 K, *R*_xx_ ~ −*α*ln(*T*); then, at *T*_K_ around 9.3 K, the *R*_xx_(*T*) emerges into the NFL regime, as shown in Figure 2c, where *R*_xx_(*T*) clearly deviates from the −*α*ln(*T*) and can be fitted well with −*βT*^1*/*2^, consistent with the orbital 2CK model. At lower temperatures below *T*_D_ of around 1 K, *R*_xx_ (T) deviates again from the −*βT*^1*/*2^, as shown in Figure 2d. One notes that the lowest temperature regime has previously never been simultaneously observed with the other two regimes since the proposal of orbital 2CK model, and the *R*_xx_ (T) of the FGaT sample can be well-described by the −*γT*^2^ down to 0.36 K as shown in Figure 2d, exactly as proposed by Zawadowski. This is the first time that all three regimes of the orbital 2CK model have been observed in a single sample, conclusively demonstrating that the resistance upturn in the FGaT is caused by the orbital 2CK effect. Furthermore, the applied magnetic field only shifts the resistance *R*_xx_ (T); it does not affect the critical temperatures of the three-resistive regimes, nor change the coefficients *α*, *β* and *γ,* of the three regimes as shown in Figure 2b,c,e. This shows that the resistance upturn is of nonmagnetic origin, consistent with the orbital 2CK model induced by the TLSs as pseudospin. After the sample was oxidized in the atmosphere for 7 days, the longitudinal resistance *R*_xx_ (*T*) increased from approximately 6 Ω to 24 Ω as shown in Figure 2d,e, but both the coefficients and (*T_D_*) remain unchanged; this indicates that the origin of the TLSs is not from the surface of the sample, and may come from defects such as grain boundaries, dislocations, twists, and point defects inside the sample, as proposed in other systems [16].

**Figure 3:**
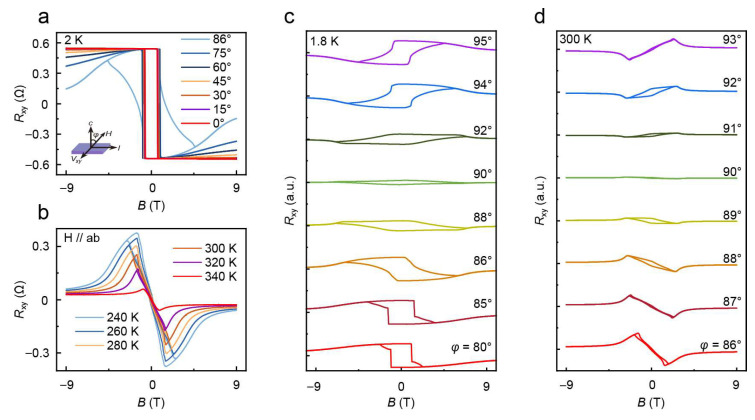
Hall effect in FGaT flakes. (**a**) *R_xy_* vs. *B*, with *H* rotating from *H*∥*c* to *ab*-plane, field up to ±9 T, *T* = 2 K. (**b**) *R_xy_* vs. *B* from 240 K to 340 K, with *H*∥*ab*, field up to ±9 T. (**c**) *R_xy_* vs. *B* for *φ* from *φ* = 80° to *φ* = 95°, field up to ±9 T, *T* = 1.8 K. (**d**) *R_xy_* vs. *B* for *φ* from *φ* = 86° to *φ* = 93°, field up to ±9 T, *T* = 300 K.

One potential method to characterize the NFL behavior is the Hall measurement [38]. We measure the Hall effect of the FGaT sample, as shown in Figure 3. At 2 K, the abrupt change in the Hall signal at the coercive field *H*_c_ suggests rapid domain flipping in FGaT, as shown in Figure 3a. As *H* rotates to the *ab*-plane, the *R_xy_* decreases and exhibits the expected angular-dependent characteristic. When *φ* = 86°, the square-shaped hysteresis loop disappears, and the magnetic domains undergo a slow flipping process towards the *ab*-plane under the magnetic field. We measure the *R_xy_* around the *ab*-plane in detail as shown in Figure 3c at 1.8 K. At *φ* = 90°, the hysteresis loop shows a small ’diamond-shaped’ structure with the largest switching field, and the Hall signal switches below and above this angle as expected for a magnetic film with perpendicular anisotropy, indicating that *φ* = 90° is near the *ab*-plane. Notably, as temperature increases from 240 K to 320 K, the Hall loop shrinks, and two antisymmetric peaks emerge in the Hall signal, as shown in Figure 3b. Antisymmetric peaks in the Hall signal have been recognized as the topological Hall signal before [39]. We measure the two peaks around the *ab*-plane from *φ* = 86° to *φ* = 93° as shown in Figure 3d. The antisymmetric peaks in the Hall signal nearly vanish at approximately *φ* = 90°, very similarly to the 1.8 K results. And the two peaks switch below and above this angle, indicating that *H* is close to alignment with the *ab*-plane near *φ* = 90°. Therefore, the observed Hall effect is simply the anomalous Hall effect of the FGaT sample. We did not find any topological Hall signal in the NFL regime of 1–9 K, and it is not conclusive that the NFL behavior can be detected via the Hall signal [39].

**Figure 4:**
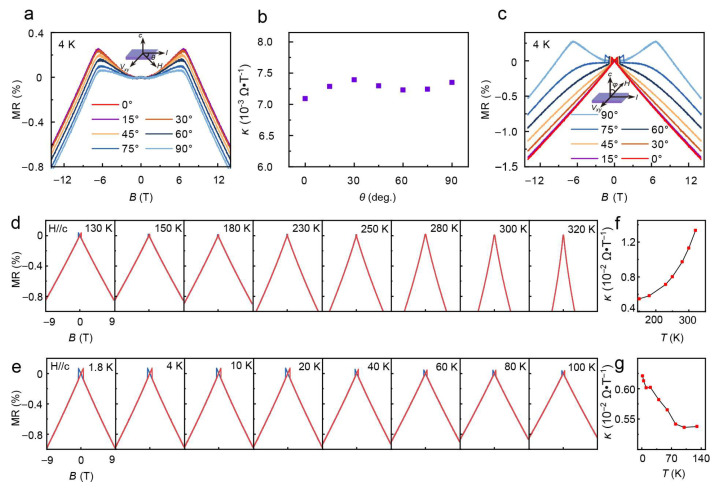
Magnetoresistance measurement of FGaT flakes. (**a**) MR vs. *B* in the *ab*-plane up to ±14 T, *T* = 4 K. (**b**) Slope magnitude *κ* from LNMR regions plotted against *θ* for *B* > 7 T, slope magnitude *κ* = |d*R*_xx_/d*B*|. (**c**) Angular-dependent MR with *H* rotating from *ab*-plane to *c*-axis, *T* = 4 K. (**d**) MR vs. *B* along the *c*-axis up to ±9 T. cooling from 100 K to 1.8 K. (**e**) MR vs. *B* along the *c*-axis up to *±*9 T, cooling from 100 K to 1.8 K. (**f**) Slope magnitude *κ* of LNMR plotted against temperature from 320 K to 130 K. (**g**) Slope magnitude *κ* of LNMR plotted against temperature from 100 K to 1.8 K.

The magnetoresistance of the FeGaT sample is measured in a field of up to 14 T in various angles, as shown in Figure 4. The magnetic field is initially aligned with the current direction and then rotated within the *ab*-plane. Over the range of −14 T to +14 T for each angle, MR exhibits two distinct dependencies on the magnetic field. When the magnetic field is less than the in-plane anisotropic field *H*_A_ (~±7 T), a quadratic characteristic with positive MR is observed. This is the expected anisotropic magnetoresistance (AMR) effect [40] since it needs a large field to bring magnetization to the field direction, consistent with the Hall measurement in Figure 3. Indeed, for field directions from the *c*-axis to the *ab*-plane, the quadratic feature in the small field becomes smaller and switches at coercive fields, as shown in Figure 3c. When the field exceeds about 7 T, the magnetization becomes saturated and aligned with the *ab*-plane, and an LNMR appears and does not saturate up to 14 T. Interferingly, the LNMR appears for the field applied in all directions and has almost the same slope, as shown in Figure 4a,c. At the same temperature, the slope magnitude *κ* of the LNMR also remains constant, as shown in Figure 4b for all directions. LNMR has been studied before and has been attributed to spin-magnon scattering in ferromagnets [41,42]. The spin-magnon scattering leads to an increase in resistance and the energy of spin waves is linearly suppressed (2*µ*_B_*B*) by an applied field, resulting in the LNMR. Therefore, the LNMR is a measurement of the spin-magnon scattering and the absolute value of the slope increases with the increasing temperature of common ferromagnets [41].

The LNMR of the FGaT samples is measured from 320 K to 1.8 K, as shown in Figure 4d,e. Strangely, the slope of the LNMR does not decrease monotonically, as depicted in Figure 4f,g. There is a minimum at about 130 K, which is very different from common ferromagnets such as Fe, Co and Ni [40]. One notes that an increase in the slope indicates a stronger spin-magnon scattering at temperatures below 130 K. This counterintuitive phenomenon suggests the presence of misalignment in the moment in this temperature range. Recently, it has been observed that there exist topological spin textures such as Skyrmion bubbles in the FGaT material between 100 K and 200 K, which may be the origin of the observed increases in the LNMR slope here [43,44,45]. Notably, the Hall signal in Figure 3 of the same sample does not show any anomaly. The spin misalignment induced by the spin texture effectively increases spin-magnon scattering.

Our work provides the first demonstration of the complete orbital 2CK model with three-resistive regimes. The Kondo regime starts at 30 K and the NFL temperature range is from 9 K to 1 K. In the 1CK effect, electrons are scattered by impurities, causing the first regime with resistance following −ln(*T*) relevance, while it becomes −*T*^2^ relevance for decreasing temperature when the impurity is fully compensated by the electron spin. The NFL behavior arises from the competition between two electrons screening an impurity. Studying this regime is important as it provides a platform for exotic physics, including phenomena related to high-temperature superconductivity, Majorana fermions, and quantum criticality [46,47,48]. The vdWs ferromagnet FGaT provides a material platform to study NFL behavior in a temperature range of 8 K with two well-defined FL regimes.

## 4. Conclusions

In summary, we have observed the full three-resistive regimes in a single FGaT sample, providing possible evidence for the orbital 2CK effect. The sequential observation of the −ln(*T*), −*T*^1/2^, and *T*^2^ scaling behaviors, along with their immunity to magnetic fields, offers evidence for this non-Fermi liquid state. The transport result indicates that the orbital 2CK effect originates from nonmagnetic TLSs. No topological Hall effect is observed in the Hall measurement, which excludes the possibility of magnetism-related NFL behavior below 30 K. The non-saturated LNMR up to 14 T reveals the spin-magnon scattering in FGaT sample. The slope of LNMR decreases non-monotonically around 130 K. Our research advances the comprehension of the orbital two-channel Kondo effect and establishes an experimental framework to study non-Fermi liquid behavior with well-defined Fermi liquid boundaries.

## Data Availability

Dataset available on request from the authors.

## Abbreviations

The following abbreviations are used in this manuscript:

2CK	Two-channel Kondo
NFL	Non-Fermi liquid
TLSs	Two-level systems
FLT	Fermi liquid theory
vdWs	van der Waals
FGeT	Fe_3_GeTe_2_
FGaT	Fe_3_GaTe_2_
*T*_C_	Curie temperature
LNMR	Linear negative magnetoresistance
XRD	X-ray diffraction
EEI	Electron–electron interaction
AMR	Anisotropic magnetoresistance
